# A Rare Case of Bradycardia and Hypotension Following Administration of Ondansetron to a Patient During Spinal Fixation Surgery

**DOI:** 10.7759/cureus.34449

**Published:** 2023-01-31

**Authors:** Sandeep Dey, Stuti Bhamri, Manish Arora, Mukesh Gupta

**Affiliations:** 1 Neuroanesthesiology and Neurocritical Care, Paras Hospital, Gurugram, IND

**Keywords:** lumbar spine surgery, prone position surgery, ondansetron, hypotension, bradycardia

## Abstract

Ondansetron is commonly used during the peri-operative period for the prophylaxis of postoperative nausea and vomiting (PONV). It is a 5-hydroxytryptamine 3 (5-HT_3_) receptor antagonist. Although relatively safe, few cases of ondansetron-induced bradycardia are described in the literature. Here, we present the case of a 41-year-old female with a burst fracture of the lumbar (L2) vertebrae following a fall from height. The patient underwent spinal fixation in the prone position. The intra-operative period was otherwise uneventful, except for an unprecedented incidence of bradycardia and hypotension following administration of intravenous (IV) ondansetron, at the time of closure of the surgical wound site. It was managed with IV atropine and fluid bolus. The patient was shifted to a intensive care unit (ICU) postoperatively. The postoperative period was uneventful, and the patient was discharged in good health on postoperative day three.

## Introduction

Bradycardia and hypotension are not uncommon under anesthesia, and the causes can vary. A few of the crucial reasons include the position of the patient (prone or sitting), extremes of either lighter or deeper plane of anesthesia, stimulation of the parasympathetic system, use of drugs (opioids, propofol, dexmedetomidine, sevoflurane, levetiracetam, etc.), and ventilation-perfusion mismatch (at extremes of hypoxia, hypercarbia, or acidosis) [1]. Most of such instances can be managed with anticholinergics, fluid boluses, and ionotropic or vasopressor agents. Though rare, complete asystole and cardiac arrest can occur, especially in aged patients, American Society of Anesthesiologists (ASA) class more than II or those with prolonged QTc interval [2]. Here, we present an unusual case of ondansetron-induced bradycardia and hypotension, very few of which are reported in the literature. 

## Case presentation

**Figure 1:**
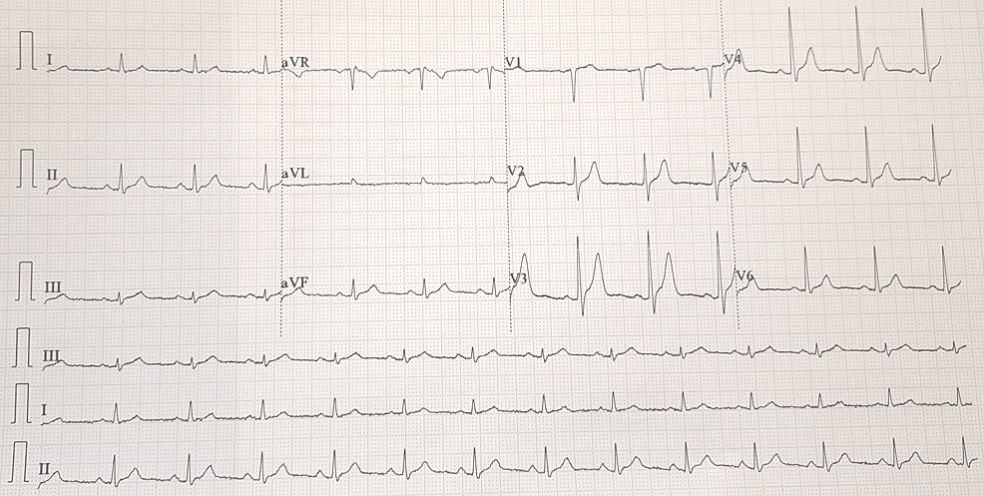
Preoperative ECG. ECG, electrocardiogram

A 41-year-old woman (56 kg) presented to our setup with an L2 burst fracture following a fall from a height of 10 feet. There was no history of loss of consciousness, vomiting, or seizure. There was no prior history of any brady or tachyarrythmia or syncopal attack. Her clinical examination and laboratory and radiological parameters were within normal limits, including a normal Glasgow Coma Scale score (15/15), pre-operative electrocardiogram (ECG) (Figure 1), 2d echocardiography, non-contrast CT brain, and cervical spine screening. She had no other known co-morbidities and was not on any medications. The patient's metabolic equivalent score was more than four and ASA physical status class II. 

On the day of surgery, the patient received tablet glycopyrrolate 2 mg as premedication. Standard ASA monitors were attached in the operation theater, and her baseline vitals were recorded. The patient was induced with fentanyl 100 mcg, propofol 100 mg, and rocuronium 50 mg. Anesthesia was maintained with oxygen and air, 2% propofol target controlled infusion (TCI) (Alaris Plus PK Care Fusion, Becton, Dickinson and Company, NJ) titrated at 2.5-4 mcg/mL using the Schnider TCI model, and ketamine infusion at 10 mcg/kg/h to maintain the Bi Spectral Index between 40 and 50. The patient was ventilated using the volume-controlled mode, with the end-tidal carbon dioxide targeted between 30 and 35 cm H2O. The patient was turned prone during the procedure. The patient's hemodynamics was stable throughout the procedure. Intraoperatively, the patient received 1300 mL of IV (isotonic) fluid along with IV paracetamol (1 g) and fentanyl (100 mcg) for analgesia.

At the time of surgical wound closure, 4 mg ondansetron was administered slowly over 1 min. This was followed by bradycardia (heart rate: 43/min) and hypotension (blood pressure: 70/38 mmHg) (Table 1). This was managed with IV atropine 0.6 mg and around 100 mL fluid bolus. This event was not concurrent with any change in the depth of anesthesia or institution of any other drug/agent known to cause bradycardia. The intra-operative arterial blood gas analysis was insignificant, and we noticed no change in the ECG following the initial episode of bradycardia. She was subsequently extubated and shifted to the ICU. We managed pain in the immediate postoperative period with fentanyl infusion. The rest of the postoperative period was uneventful, and the patient was discharged home in good health on postoperative day three. 

**Table 1:** Intraoperative hemodynamic parameters showing bradycardia and hypotension. HR, heart rate; PR, pulse rate; ART-SYS, systolic blood pressure (arterial); ART-DIA, diastolic blood pressure (arterial); ART-MEAN, mean blood pressure (arterial)

	11:17	11:18	11:19	11:20	11:21	11:22	11:23	11:24	11:25	11:26	11:27
HR (bpm)	67	58	53	48	52	46	47	43	46	61	74
PR (/min)	67	58	52	48	51	46	47	43	45	61	74
SpO_2_	100	100	100	99	99	99	99	99	99	99	99
ART-SYS (mmHg)	128	121	119	112	105	99	70	84	93	117	133
ART-DIA (mmHg)	73	66	58	54	53	52	38	45	48	61	77
ART-MEAN (mmHg)	91	84	78	73	70	68	49	58	63	80	96
Drug administered	Ondansetron 4 mg							Atropine 0.6 mg			

## Discussion

Safe anesthesia is pertinent to any anesthetic procedure and technique. However, as with any technique, general anesthesia can have its complication/s, and postoperative nausea and vomiting (PONV) is one such. The prevalence of PONV can be as high as 27.7% during the first 24 h, and different classes of drugs are used to prevent it [3]. Selective 5-hydroxytryptamine 3 (5-HT3) (serotonin) receptor antagonists like ondansetron are amongst the most commonly used drugs for prophylaxis of PONV. 

Ondansetron blocks serotonin receptors both in the gut (peripherally) and in the chemoreceptor trigger zone and nucleus tractus solitaries (centrally). It is safe, effective, and lacks significant drug interaction. However, there are reports of QT prolongation observed with ondansetron, particularly in patients with congenital long QT syndrome, electrolyte abnormality (hypokalemia, hypomagnesemia), congestive heart failure, or concomitant use of drugs that increase the QT interval [4]. Other rare side effects reported with ondansetron include fatal ventricular tachycardia, atrial fibrillation, and severe bradycardia with respiratory arrest [5-6].

The bradycardia associated with ondansetron is attributed to its blocking of "human ether a-go-go related gene (HERG)" encoded potassium (K+) channels, prolonging repolarization and action potential plateau phase [7]. Other 5-HT3 receptor antagonists like palonosetron block HERG encoded Na+ and K+ channels and can lead to ventricular arrhythmia. It is also postulated that activation of 5-HT3 receptors in coronary vasculature stimulates the Von Bezold Jarisch (VBJ) reflex leading to apnea, bradycardia, hypotension, and blockage of the 5-HT3 receptors can lead to tachyarrhythmia. Thus in any patient, the effect of a 5-HT3 receptor antagonist like ondansetron is dependent upon the pre-existing serotonergic activity of the autonomic nervous system [8].

Only six cases of ondansetron-induced bradycardia have been described in the literature so far [9-10]. In our case, ondansetron-induced bradycardia followed by hypotension was observed in a 41-year-old female undergoing spinal fixation for traumatic L2 vertebrae burst fracture. There was a decrease in the heart rate (HR) and blod pressure (BP) from 74-78/min to 43/min and 110-116/76-80 to 70/38 mmHg, respectively that was managed with IV atropine 0.6 mg and fluid bolus. The rest of the postoperative period was uneventful, and the patient was discharged home in good health. In our case, the Naranjo adverse drug reaction probability score was four, indicating a possible association between the reaction (bradycardia and hypotension) and the drug (ondansetron) [11]. 

Bradycardia and hypotension are salient intraoperative concerns and can lead to grave patient-related catastrophes if not treated in time. It is of paramount importance in neuroanesthesia and neurocritical care as the patient can have many risk factors that can predispose them to bradycardia, like position (prone or sitting), drugs used (opioids, levetiracetam, dexmedetomidine), raised intra cerebral pressure, or electrolyte abnormalities [1, 4, 6]. Slow administration over two to five minutes is highly effective in decreasing the incidence of PONV in a susceptible patient population while avoiding its rare adverse effect like bradycardia [12]. Alternatively, one can use other safer alternative like palonosetron, which has a longer duration of action and is devoid of QT prolonging effect in weight-adjusted dose and in the absence of other precipitating factors.

## Conclusions

Ondansetron is one of the most commonly used antiemetics for PONV. It is safe, potent, cost-effective, and without significant drug interactions. There are rare reports of bradycardia progressing to atrial or ventricular tachycardia or even asystole and apnea following IV ondansetron administration. Though rare, clinicians should be aware of such complications, be cautious about administering the drug very slowly, and have a keen eye for observation post-administration to deal with any adverse drug reaction.
